# Ultrasound guided puncture of the radial artery for blood gas analysis: a prospective, randomized controlled trial

**DOI:** 10.1186/1757-7241-23-S1-A16

**Published:** 2015-07-16

**Authors:** Christian B Laursen, Rasmus L Pedersen, Annmarie T Lassen

**Affiliations:** 1 Department of Respiratory Medicine, OUH Odense University Hospital, Odense, Denmark

## Background

Arterial puncture for arterial blood gas analysis (ABGA) is a procedure often performed in the emergency department (ED). Using ultrasound (US) guidance of the procedure as a routine could potentially increase the proportion of patients in which arterial puncture for ABGA is successful in the first attempt.

## Methods

A prospective, parallel-group, randomized controlled trial was conducted in an emergency department. Patients being admitted to the ED or already admitted to the ED were included in the study if the physician attending the patient ordered an ABGA. Exclusion criteria were permanent mental disability, patient age < 18 years, patients declining to participate in the study or ABGA contraindicated. Patients were randomly assigned to arterial puncture using the standard procedure, or to US guided puncture. The primary endpoint of the study was the proportion of patients in which arterial puncture for ABGA was successful in the first attempt.

## Results

238 patients were included and randomized. 115 patients remained for analysis in the US group and 109 remained in the control group. The proportion of patients in which arterial puncture for ABGA was successful in the first attempt in the US group was the proportion of patients was 103 (89.6%), versus 103 (94.5%) in the control group (p = 0.18). The absolute and relative effect were -4.9% (95% CI: -12.5 to 2.5) and 0.95 (95% CI: 0.90-1.06) respectively.

## Conclusion

In an emergency department setting, the routine use of US guided arterial puncture does not increase the proportion of patients in which arterial puncture for ABGA is successful in the first attempt, when compared to ABGA obtained by the conventional technique.

## Trial registration

Registered at http://clinicaltrials.govNCT01660724.

